# FreiLaser: Flexible pulse generator for laser control in optogenetic experiments

**DOI:** 10.1016/j.ohx.2025.e00709

**Published:** 2025-10-04

**Authors:** Artur Schneider, Ilka Diester

**Affiliations:** a Optophysiology Lab, Institute of Biology III, University of Freiburg, Freiburg, Germany; b IMBIT//BrainLinks-BrainTools, University of Freiburg, Freiburg, Germany; c Bernstein Center for Computational Neuroscience, University of Freiburg, Freiburg, Germany

**Keywords:** Optogenetics, Laser, Neuroscience, Circuitpython, Microcontroller, Behavior

## Abstract

To facilitate optogenetic experiments in neuroscience, we designed a cost-effective (<40 €) and versatile laser control system, FreiLaser, based on the Raspberry Pi Pico microcontroller running CircuitPython. FreiLaser allows precise control over various stimulation parameters for up to four lasers, utilizing both analog and digital signals. It features a user-friendly graphical interface for parameter configuration and real-time visualization, as well as an API for seamless integration with existing experimental setups. The system also includes a built-in mask controller to prevent behavioral bias by synchronizing masking LEDs with laser pulses. Validation tests confirmed that FreiLaser generates stable, temporally precise control signals, suitable for a range of stimulation patterns. The system’s flexibility, ease of use, and low cost make it an invaluable tool for researchers conducting optogenetic and behavioral studies. Our open-source design ensures accessibility and adaptability for a wide range of experimental needs.

## Graphical abstract

**Figure:**
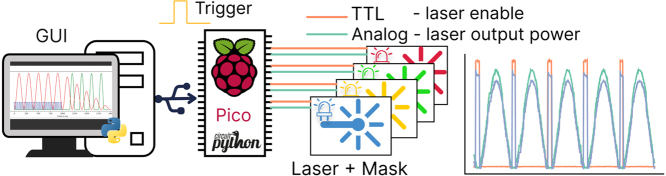

## Specifications table

**Table:** 

Hardware name	FreiLaser
Subject area	Neuroscience
Hardware type	Microcontroller for pulse generation
Closest commercial analog	PulsePal https://sanworks.io/shop/viewproduct?productID=1102
Open source license	GNU General Public License v3.0

Cost of hardware	∼40 € for base elements, plus LED-elements depending on requirements total <280 € for four masking colors.

Source file repository	https://github.com/arturoptophys/FreiCtrl_Laser
	https://doi.org/10.5281/zenodo.15682570

	

## 1 Hardware in context

Optogenetics has become a widely used tool in neuroscience for temporally and spatially precise modulation of neural activity, facilitating the establishment of causal relationships [1]. This technique involves the manipulation of the activity of genetically engineered, typically electrically conducting cells through the use of light-sensitive membrane proteins known as opsins. These opsins can be ion channels (e.g., channelrhodopsin-2 (ChR2) [2], [3]), pumps (e.g., halorhodopsin (NphR) [4], [5]), or G protein-coupled receptors (e.g., eOPN3 [6]). The effects of opsins on the neurons that express them are diverse, including: (1) depolarization of the membrane potential, leading to activation [3]; (2) hyperpolarization, leading to silencing [5]; and (3) modulation of vesicle trafficking at the synapse, leading to presynaptic inhibition [6]. These tools have been successfully used not only in cultured cells but also in freely behaving rodents [7], [8] and in various mammals, including primates [9].

In recent years, many new and modified opsins have been developed to improve their properties and adapt them to different tasks. Shifting the activation wavelength to the red/far-red spectrum [10] not only allows for deeper tissue penetration, but also facilitates the modulation of larger volumes due to the reduced absorption of red-shifted light. Typically, light is delivered to the brain tissue via optical fibers [11], [12]. These fibers are coupled to the light source, which can be an LED or, preferably, a laser for precise wavelength and temporal control.

Current experimental approaches often require the use of different opsins, each activated by different wavelengths. Innovative technologies such as BiPOLES allow bidirectional control of neuronal activity by integrating an inhibitory opsin with an activating opsin, each responsive to different wavelengths [13]. Similarly, the bistable PdCO facilitates prolonged presynaptic inhibition, allowing researchers to initiate and terminate inhibition with different wavelengths [14]. In summary, experiments may require flexible multi-wavelength control and adaptable parameterization.

Even when single opsins are used, the stimulation parameters chosen may vary depending on the experimental goals. Researchers must determine the optimal stimulation frequency, pattern, light intensity, and timing to use [15]. Some protocols distinguish between continuous and pulsed stimulation patterns, each of which affects neuronal firing and behavioral responses differently. Continuous patterns tend to induce reliable, sustained currents and neuronal spiking, but with less temporal precision in spike timing [3] and potentially significant heating issues [16]. In addition, the chosen stimulation power and duty cycle critically affect the total energy delivered and consequently the temperature rise. For pulsed stimulation, both sinusoidal and square-wave patterns are possible. Sinusoidal patterns can minimize photoelectric artifacts and allow phase-locking of neural responses [17]. Crucially, the choice of stimulation pattern also influences the behavioral effects of stimulation [18].

Different stimulation frequencies can be used to replicate specific neural rhythms or oscillations [15]. In addition, varying the frequency for the same population of neurons can result in different behavioral outcomes [19]. Similarly, the timing of stimulation is critical to fully exploit the temporal resolution of optogenetics. Perturbations can be precisely timed to the millisecond, allowing interference with specific neural processes [15]. Moreover, only stimulations that are temporally aligned with behavioral events are likely to have behaviorally relevant effects [20].

In summary, there is a significant need for hardware that provides temporally precise control of optogenetic perturbations, along with flexible parameter selection and ease of use for scientists performing optogenetic experiments. While commercially available systems such as TDT or MED Associates offer customizable output channels for controlling laser output, these systems are expensive, often closed-source, and lack customization options. The development of more affordable, open-source tools such as PulsePal (https://github.com/sanworks/PulsePal) has led to their widespread adoption in laboratories. Open-source solutions for Optogenetics TTL driver based on Arduino hardware (https://open-neuroscience.com/en/post/optogenetics_ttl_driver/) exist; however, they offer limited customizability and only digital output signals (i.e., no sinusoidal stimulation).

Here, we present a flexible, low-cost system (<40 €) based on the Raspberry Pi Pico microcontroller board running CircuitPython. Designed for use in optogenetic experiments within behavioral paradigms, it can control up to four lasers simultaneously through both analog and digital channels. Wide range of stimulation parameters can be flexibly configured via an intuitive graphical user interface (GUI) or an Application Programming Interface (API). The system’s multiple triggering options provide extensive adaptability to different experimental setups. In addition, a built-in mask controller automatically generates signals to drive masking LEDs, preventing animals from associating the laser with a visual cue. FreiLaser can operate independently or be seamlessly integrated into existing experimental systems.

## 2 Hardware description

• Low-cost signal generator for laser control, tailored for optogenetic experiments.
• Supports multiple waveforms, including square, sinusoidal, and half-sinusoidal.
• Offers a wide range of parameters configurable via a user-friendly GUI or a serial API.
• Includes six selectable trigger inputs and a priming pin to ensure correct triggering.
• Provides temporal precision suitable for behavioral experiments.
• Software written in CircuitPython, offering high customizability.

**Fig. 1:**
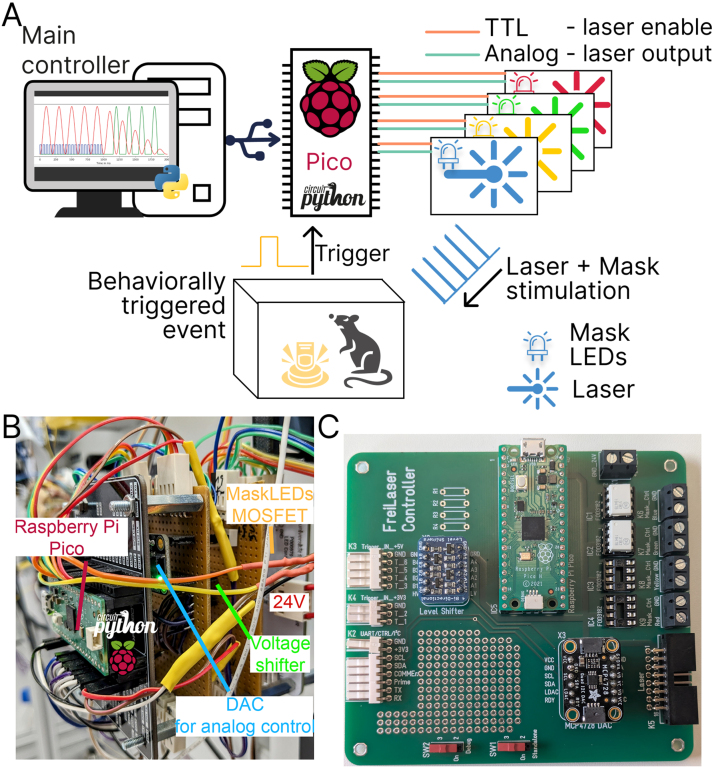
Overview of the FreiLaser system. **A:** FreiLaser is constructed around a Raspberry Pi Pico microcontroller running CircuitPython. Stimulation parameters can be adjusted via a GUI. Upon the occurrence of a software or hardware trigger signal, the Pico initiates the generation of pulses. The laser output is modulated via a TTL signal, which controls the laser enable, and an analog signal, which controls the laser output power. Masking LEDs are trigger in the same pattern as lasers. **B:** Prototyping version of the FreiLaser system. TTL-like signals are produced by the Pico itself, and the analog signals via a digital–analog-conversion (DAC) breakout board MCP4728A4. Voltage differences between 3.3 V (Pico) and 5 V (e.g. arduino elements) are adjusted using a voltage shifter breakout board Adafruit 757. MOSFETs drive the masking LEDs based on control signals from Pico. **C:** Assembled PCB for the FreiLaser. Please note resistors R1-4 and IC3-4 were not installed when taking this image.

The FreiLaser system is a cost-effective signal generator designed for the control of tabletop lasers in optogenetic applications within behavioral settings. The system incorporates a Raspberry Pi Pico microcontroller programmed in CircuitPython, complemented by circuit for generating analog signals (12-bit resolution). Moreover, the system incorporates circuits for driving ambient LEDs for masking purposes (Fig. 1**A,B,C**). FreiLaser is accompanied by a PyQt6-based GUI, which allows users to set various stimulation parameters and visualize the resulting signals (see Table 1).

**Table 1:** Feature overview.

Feature	Specification
Design	PCB or breadboard
Microcontroller	Raspberry Pi Pico (CircuitPython)
Output channels	Up to 4 lasers + 4 masking LED channels
Analog output	0–5 V, 12 bit resolution
Digital output	3.3 V
Waveforms	Square, sinusoidal, half-sinusoidal
Attenuation	For sinusoidal, half-sinusoidal, ramp down
Inputs	6 selectable (2x 3.3 V direct, 4x 5 V via level shifter) triggers
Other inputs	Priming pin
Max frequency tested	200 Hz (square); 50 Hz (sine)
Min pulse duration tested	≥2 ms
Trigger inputs	6 selectable (3.3 V direct, 5 V via level shifter)
Masking LEDs	Up to 4 channels, 24 V LED strips (synchronized with lasers)
Software	PyQt6 GUI, Serial API

	Operational modes

Standalone	Parameters and triggers set via GUI
Semi-standalone	Parameters from GUI; triggers from external hardware
Autonomous	Parameters, triggers, priming via external system (e.g., behavioral controller)

FreiLaser can manage up to four lasers simultaneously, leveraging both analog and digital TTL-like signals for each. Tabletop diode lasers (Omicron LuxX series, Coherent OBIS, CNI FC-W-X series, OptoEngine MBL-H), can be controlled using an analog control input to adjust output power and a digital signal to toggle laser emission. By combining these control signals, complex stimulation patterns can be achieved. Up to four lasers (each with their own parameters) can be triggered simultaneously (or delayed via parameters) using a single trigger signal. The mask circuit also enables simultaneous or independent control of ambient light flashes, mirroring the parameters of laser stimulation. Employing such masking techniques helps prevent animals from associating laser stimulation with behavioral cues [21].

**Fig. 2:**
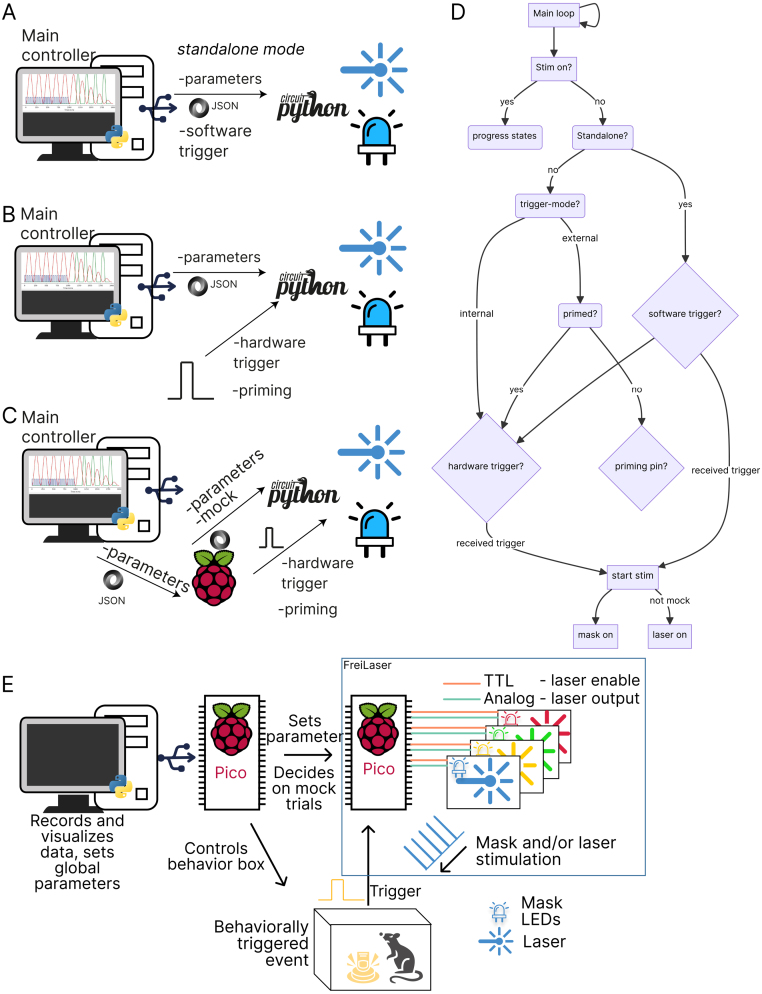
Overview of the operational modes. **A:** Standalone mode. Parameters and trigger signals are send via GUI. **B:** GUI-controlled parameters with external trigger. Trigger signals are generated by further hardware elements. **C:** Fully autonomous mode. Parameters are transmitted from other systems via API. **D:** Logic flowchart for the main loop of FreiLaser. **E:** Diagram indicating components of a fully integrated FreiLaser system in fully autonomous mode. Additional microcontroller, which is also controlling the behavioral box, transmits the parameters to FreiLaser as well as decides which trials should be stimulated based on behavior and settings from main computer.

The parameters (i.e., the specific lasers, masks, and stimulation parameters to be utilized) can be transmitted from the GUI or API via universal serial bus (USB) serial or the universal asynchronous receiver/transmitter (UART) input (Fig. 2**A**). This determination is made via a standalone switch (Fig. 4**A**). Following each stimulation event, the parameters may be modified in a flexible manner. A variety of inputs can be selected as trigger signals. Directly wired signals should be 3.3 V. The remaining inputs are wired through a voltage shifter, allowing for inputs of 5 V (or other voltages) to be used.

FreiLaser supports multiple operational modes, each designed to accommodate different experimental requirements:

• Standalone mode — Fig. 2**A** In this mode, all parameters and triggers are controlled directly from the GUI without the need for additional hardware. This is the simplest mode to implement and is ideal for straightforward experimental setups.
• Semi-standalone mode — Fig. 2**B** Parameters are set via the GUI, but triggering and, optionally, priming signals are generated by external hardware. This mode offers a balance between control and integration with other experimental apparatus.
• Autonomous mode — Fig. 2**C** In this most flexible mode, external devices (e.g., another Raspberry Pi Pico controlling the behavioral setup Fig. 2**E** [22]) manage or transfer parameters to FreiLaser via the API. Both trigger and priming signals are also generated by external hardware. This mode allows deep integration into existing systems and high flexibility.

**Fig. 3:**
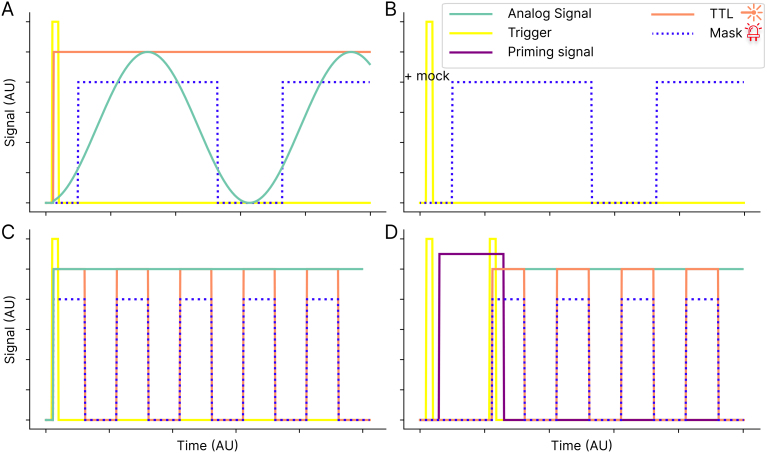
Overview of the temporal profile of FreiLaser function. **A:** A preset stimulation pattern (e.g. sinosiodal wave) is executed upon a trigger signal being detected. In standalone mode this trigger is a software signal. FreiLaser simultaneously controls analog and TTL signal to drive the desired laser output, combined with synchronized mask signal driving masking LEDs. **B:** If a mock software signal is received, only the mask signal is produced upon trigger. **C:** Similar as in A, but with a squarewave signal. Now the TTL is modulated and analog output remains stable. **D:** Visualization of the priming mode. The first trigger signal which arrived without the priming signal does not start stimulation. Only when trigger and priming signals coincide, the stimulation is started.

Further inputs in the FreiLaser system include a priming feature for the trigger, specifically designed to address potential unreliability in trigger signals, such as those from beam breakers. Trigger signals may occasionally be unstable, triggering multiple times per trial—for example, if an animal repeatedly crosses a beam back and forth or activates the trigger outside of the designated task phase, such as during the inter-trial interval. To mitigate this issue, FreiLaser employs a priming pin. This pin opens a reporting window, such that only trigger signals coinciding with the priming signal initiate stimulation (Figs. 2**D**, 3**D**). In this procedure, a HIGH signal on the priming pin sets the FreiLaser in the primed(ready) state, such that the next trigger signal will start the stimulation. Repeated trigger signals without a new priming signal will not initiate stimulation. Thus, a stimulation may be set to occur only once per trial or during a specific time window.

**Fig. 4:**
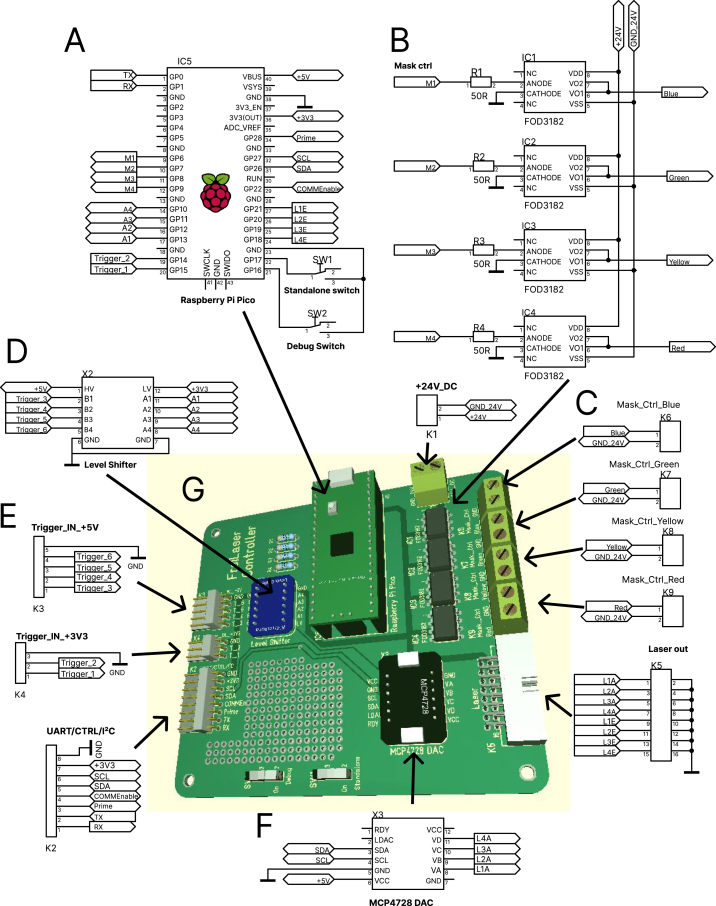
Connection diagram of the FreiLaser system. **A:** Raspberry Pico as main controller. **B:** Mask signals mimicking the laser output are generated internally and sent to the mask circuit. Mask circuit controls the LED output to mimic the laser output via ambient light. FOD3182 optoisolated gate drivers are used to control the LED output via 24 V power supply. **C:** Four independent circuit can be used to mask up to four different laser wavelengths. **D:** Adafruit 757 is used for shifting the input levels from 5 V to 3.3 V **E:** Signal inputs. **F:** MCP4728A4 is used as DAC to generate analog signals to control the laser output power. **G:** PCB for the FreiLaser, for more details see design_files/Laser_Control_PCB.pdf in the repository.

FreiLaser leverages CircuitPython, a microcontroller-adapted version of Python, offering an accessible programming environment with a comprehensive array of available libraries. The platform facilitates the direct transfer of data between the board and a computer via standard libraries, including those for serial communication and JSON parsing. The object-oriented programming approach facilitates the implementation of control elements in a state-machine-like structure, enhancing the system’s modularity and maintainability. Interactive coding through CircuitPython’s prompt significantly simplifies prototyping and debugging, making it a more user-friendly option for beginners compared to traditional Arduino C++ programming.

The core of FreiLaser’s functionality resides in its main loop. During each cycle, the system checks if a stimulation is active and adjusts its outputs accordingly, progressing through its states (Fig. 2**D**). Each laser is managed by an instance of the “LaserController” class defined in *laser_daq.py*, which holds its specific parameters and state, and controls the timing for each laser independently. When no stimulation is active, FreiLaser monitors the serial inputs for new parameters or software triggers. It then watches the designated trigger channels and initiates stimulation if a trigger — and optionally, a priming signal — is detected.

Limitations of the FreiLaser system:

• FreiLaser was designed for laser systems which are modulated via a combination of analog and digital signals and thus may required modification for use with other lasers.
• FreiLaser might not be suitable for use cases (e.g. spike-triggered stimulation) where a maximally short lag (μs range) is desired.
• Stimulation frequencies above 200 Hz have not been tested.
• Stimulation frequencies above 50 Hz for sine stimulation have not been tested.
• Pulses shorter than 2 ms have not been tested.
• The output TTL signals are 3.3 V and may be incompatible with some systems that expect a different voltage.
• Trigger signals can be both TTL and analog signals, however, only signals going from HIGH to LOW will be detected as trigger signals. There is no possibility of relaying an analog control signal to the laser output. For such scenarios, users may want to directly connect their desired analog control signal to the laser and use FreiLaser to control the laser-enable on-off states.

## 3 Design files summary

See Table 2.

**Table 2:** List of design files.

Design filename	File type	Open source license	Location of the file
design_files/ Laser_Control_PCB.zip	Gerber files	GNU General Public License v3.0	https://github.com/arturoptophys/FreiCtrl_Laser

design_files/ Laser_Control_PCB.pdf	Circuit design	GNU General Public License v3.0	https://github.com/arturoptophys/FreiCtrl_Laser

design_files/ Laser_Control_PCB.T3001	Circuit design	GNU General Public License v3.0	https://github.com/arturoptophys/FreiCtrl_Laser

FreiCtrl_Laser repository	Code	GNU General Public License v3.0	Available with the article and https://github.com/arturoptophys/FreiCtrl_Laser

circuitpython code	Code	GNU General Public License v3.0	Available with the article and https://github.com/arturoptophys/FreiCtrl_Laser

setup.py	Code	GNU General Public License v3.0	Available with the article and https://github.com/arturoptophys/FreiCtrl_Laser

• design_files/Laser_Control_PCB.zip — Gerber files for PCB production.
• design_files/Laser_Control_PCB.pdf — Wiring diagram of the FreiLaser as in Fig. 4.
• design_files/Laser_Control_PCB.T3001 — Circuit design technical file, can be viewed with free Target3001! software.
• FreiCtrl_Laser — folder containing the code required to run the GUI. Contains *GUI_laser.py* as the main file to be run. Further files contain necessary utility functions, and GUI design files.
• circuitpython code — folder containing code for circuitpython on pico. *main.py* is the main code being run. *boot.py* is code being run once at boot time. *timing_utils.py* and *laser_dac.py* contain further utility functions.
• setup.py — python install file to install the FreiCtrl_Laser and its requirements as module (see Table 2, Table 3).

**Table 3:** List of components with price and sources.

Designator	Component	Number	Cost per unit - currency	Total cost - currency	Source of materials	Material type
PCB	Printed circuit board	1.	16.34 €	16.34 €		Other
IC5 (Pico)	Raspberry Pi Pico H	1	4.6 €	4.6 €	DIGIKEY 2648-SC0917-ND	Micro-controller
X3 (DAC)	MCP4728A4/Adafruit 4470	1	6.9 €	6.9 €	DIGIKEY 1528-4470-ND	Semiconductor
X2 (Voltage shifter)	Adafruit 757	1	3.63 €	3.63 €	DIGIKEY 1528-1007-ND	Semiconductor
SW1,2	switch	2	1.11 €	2.22 €	RS-Components 913-9065	Other
K2	8-pin	1	0.75 €	0.75 €	RS-Components 679-5482	Other
K3	5-pin	1	0.70 €	0.70 €	RS-Components 679-5460	Other
K4	3-pin	1	0.39 €	0.39 €	RS-Components 679-5451P	Other
K5	16-pin Box connector	1	0.23 €	0.23 €	Reichelt WSL 16W	Other
K1,K6–9	terminal strip	5	0.24 €	1.20 €	Reichelt AKL 101-02	Other

Optional mask components						

IC1–4	FOD3182	0–4	2.91 €	11.64 €	DIGIKEY FOD3182-ND	Semiconductor
IC1–4	DIP-8 socket	0–4	0.19 €	0.76 €	DIGIKEY 1-2199298-2	Other
R1–4	Resistor 50 Ω	0–4	0.09 €	0.36 €	DIGIKEY 13-MFR25SF BE52-50R-ND	Semiconductor
LED-stripes	Barthelme LED strip color	0–4	45.37 €	45.37 €	Conrad.de 2568619 - 62	Other
24 V LED driver	Meanwell HLG-100H-24	0–1	55.75 €	55.75 €	DIGIKEY 1866-2300-ND	Other

## 4 Bill of materials summary

See Table 3.

## 5 Build instructions

Warning: electrical hazard — risk of electric shock. Always de-energize circuits before working, wear appropriate protective gear, and ensure electrical equipment is operated according to its specifications with correct voltage and current settings.

### 5.1 Hardware

#### 5.1.1 Main circuit — using the PCB

**Fig. 5:**
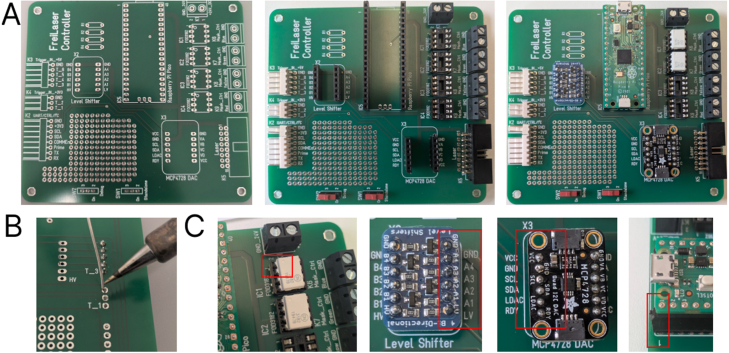
Building the PCB **A:** Solder the connectors, sockets, and headers to the board. **B:** Ensure proper solder flow for good connections. **C:** Insert the breakout boards, pico and the FOD2182 in the indicated orientation. Red squares indicate how to orient the parts. (For interpretation of the references to color in this figure legend, the reader is referred to the web version of this article.)

We provide a PCB design for ease of assembly. Gerber files (design_files/Laser_Control_PCB.zip) are available for PCB production at https://jlcpcb.com/ or https://portal.multi-circuit-boards.eu. On the PCB only the connectors and few elements need to be soldered. Headers can be added for the breakout boards and the Pico to allow easy replacement. Similarly, the FOD3182 should be installed using DIP-8 sockets (see Fig. 5).

#### 5.1.2 Main circuit — manual wiring

The circuits for the FreiLaser system can be wired on a breadboard for simplicity and testing purposes. In addition to the components listed in the bill of materials, soldering equipment or solderless breadboards are necessary. Wire the individual components according to Fig. 4.

• Adafruit 757(X2) voltage shifter is required if any components sending a trigger signal operate on other voltage than 3.3 V.
• In our case some trigger signals (Trigger_IN+5V) come from beam-breakers which operate on 5 V, thus the Adafruit 757 is wired to 5 V as high voltage (HV-pin). Please adopt the HV according to your needs.
• The DAC (MCP4728A4, X3) allows to output complex waveforms or control the laser output power via an analog signal. VCC-pin is wired to 5 V line as we would like our analog signal to range between 0–5 V. If other ranges are required, wire the VCC accordingly.
• The DAC is connected to the pico via I2C-protocol which enables fast control of the output.
• The Pico can be powered via the USB connection or a 5 V input to the VBUS pin. WARNING: do not connect the VBUS if pico is powered via USB.
• Debug switch — switch to mount pico’s drive as a file system. If connected to GND, files can be modified/copied to the board. If not connected, the file system is mounted internally for logging.
• Standalone switch — switch to operate FreiLaser in standalone mode (parameters via USB) or via UART. If pin is connected to GND, FreiLaser operates in standalone mode (Fig. 2**A**).
• Prime pin — connect to the source of the priming signal. 3.3 V only, otherwise wire through Adafruit 757.
• CommEnable pin — Indicates whether the system is in an idle state and ready to receive new parameters. If HIGH, pico does not accept new parameters and only awaits trigger signals. Connect this pin to GND if this functionality is not required. This is disabled in standalone mode.
• Connect each laser’s analog control (L1A to L4A) and laser enable signals (L1E to L4E) to your laser’s corresponding channels.
• Connect the mask outputs (M1-4) to the masking circuit as required.

#### 5.1.3 Masking circuit

The masking circuit is an optional component of the FreiLaser system, utilized primarily in behavioral experiments where there is a risk that animals might perceive the laser light and use it as a cue. It is designed to drive LEDs to produce light flashes synchronized with laser pulses, effectively masking any potential visual cues from the lasers. It uses FOD3182 optocouplers driver (prefaced with 50 Ω resistors to limit the current) to switch the LEDs Fig. 4**B**.

We employ 24 V LED strips in red and blue to match the corresponding laser colors. The number and length of LED strips required depend on the size of the behavioral setup. For a large area measuring approximately 2 × 1 m, we utilized four strips of approximately 20 cm each per color [22]. It is crucial to ensure sufficient illumination to effectively mask the laser throughout the entire experimental area. Wire the LED stripes, the FOD3182 optoisolated gate driver and 24 V power source according to Fig. 4**B** or use the PCB.

### 5.2 Software

#### 5.2.1 CircuitPython

Please install CircuitPython firmware on your pico board according to instructions (https://circuitpython.org/board/raspberry_pi_pico/). In short: download the *.uf2 firmware, press BOOTSEL button on the board while plugging it into USB. It will appear as a USB disk drive, copy the *.uf2 firmware on it. The board will reboot and is now ready to execute CircuitPython code. Functionality was tested on CircuitPython versions ≥7.XX and ≤8.X.X. The board will appear as “CircuitPython” drive on your computer. Now you can copy the code files (from circuit_python_code folder) on to the drive. Now the LED on the pico should blink with 6 Hz indicating its functioning properly. If changes to the code are required see notes about the Debug switch.

#### 5.2.2 GUI

**Fig. 6:**
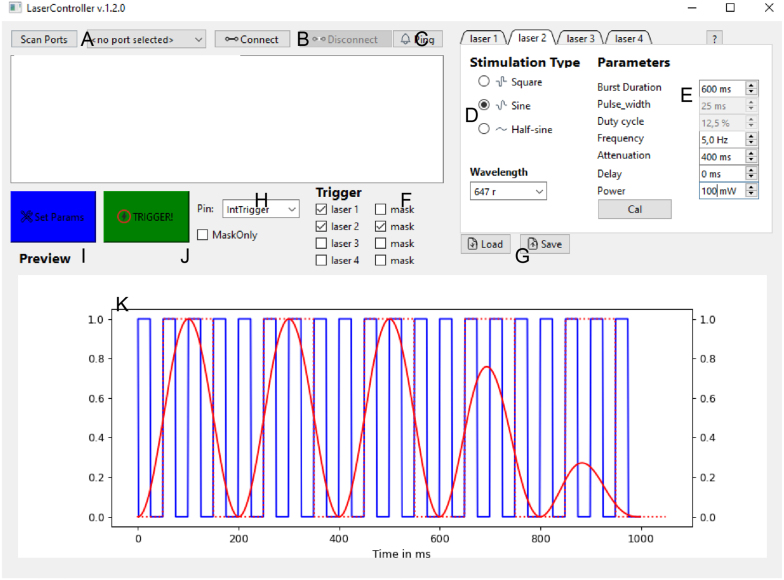
FreiLaser GUI. **A:** Scan serial ports. Choose the data-serial port of the pico from dropdown. Under Linux typically ttyACM1, windows some COM-port. **B:** Connect/disconnect to the chosen serial port. **C:** Ping board. Sends a echo request, turns green if board responds. **D:** Per laser parameters. Waveform options, wavelength determines the visualization color. **E:** Various parameters of the stimulation. Some options are only available for specific stimulation waveforms. **F:** Choose active lasers and/or masks **G:** Save/load settings to/from file. **H:** Dropdown of the trigger-input channel. **I:** Send current parameters to the board. **J:** Send software trigger signal to the board. This will trigger laser stimulation according to parameters set before! **K:** Visualization of the current parameters. Filled line indicate the laser output and skipped lines the mask.

To use GUI (see Fig. 6) for visualization and setting of the parameters for the FreiLaser you need to install the dependencies. We recommend installing the GUI inside a conda/virtual environment.

**Figure:**
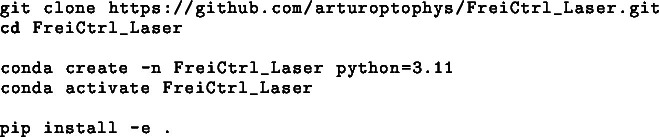

Start the GUI with from within the virtual environment:

**Figure:**
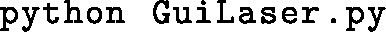

## 6 Operation instructions

Warning: Laser radiation is harmful to eyes and skin. Avoid direct eye exposure, and wear protective clothing and goggles when working with lasers!

Typical operation procedure (Fig. 2**D**):

• Set the desired parameters via the GUI or API.
• (optional) Set the mock signal if necessary.
• (optional) Drive the prime pin HIGH if using priming.
• Drive the trigger pin HIGH to start stimulation.
• Stimulation will continue according to the set parameters.
• Set the trigger and prime pins to LOW to enable the next trigger.
• (optional) Set new parameters if further adjustments are needed.

For the simple case of using the same stimulation parameters, those only need to be set once. The trigger signal can then be delivered repeatedly to initiate stimulations. The trigger signal is debounced for 5 ms (modifiable via the *TRIGGER_DEBOUNCE* variable) to prevent spurious signals from triggering the stimulation.

FreiLaser enables both laser+mask and mask-only stimulations Fig. 3**A,B**. In this manner, some trials can be implemented as controls, whereby no laser stimulus is delivered. This “mock” or null signal is sent to the FreiLaser via the API and can be modified after each stimulation. Thus, an external system can decide (systematically or randomly) which trials should contain a laser stimulus and which should be used as controls.

### 6.1 Stimulation parameters

Stimulation types:

• Square — TTL-like square wave signal. The amplitude, frequency and duty-cycle can be varied.
• Sinusoidal — slowly changing signal. The amplitude and frequency can be varied.
• Half-sinusoidal — only upper part of the sinusoidal, with signal going to 0 for half-phase.

Further parameters:

• Burst duration: Total duration of the pulse train in milliseconds (ms).
• Pulse width (square only): Duration of a single pulse in milliseconds (ms).
• Duty cycle (square only): Duration of a single pulse in relation to its phase-duration.
• Frequency — Frequency of the pulse train (Hz).
• Attenuation — (sinusoidal): Duration (ms) over which the pulse train continues while ramping down the amplitude to 0.
• Delay : Delay (ms) between the trigger and the start of the pulse train. This can be used to offset a pulse train relative to others.
• Power: If calibrated, the desired output power (mW); otherwise, in percentage (%) of the maximum laser output.

### 6.2 GUI

We provide a GUI based on Python and PyQt6 which allows setting the parameters and visualizing the resulting stimulation patterns (Fig. 6). GUI manages the connection to the pico board via USB-serial (Fig. 6**A,B**). In the GUI all the parameters (Fig. 6**E,I**) described before can be set, as well as the trigger-input (Fig. 6**H**) and which lasers and /or masks should be used (Fig. 6**D**). With the GUI, FreiLaser can be used in the standalone mode (Fig. 2**A**), hereby no further hardware is required. The lasers can be triggered via a software signal from the GUI (Fig. 2**J**).

The GUI also supports laser output calibration for individual lasers. FreiLaser will trigger multiple 10 s pulses at different intensities (defined in the *CALIB_STEPS* variable). The laser output can be measured using an optical power meter, and the values can be entered into the corresponding fields in the command line. The GUI will then calculate a linear regression from the entered values and save the data to a JSON file, enabling the system to scale the laser output accordingly. After calibration, the power values of the laser can be entered in mW, and FreiLaser will scale the control signal to match these values. Warning: Ensure that your laser has an approximately linear response curve, and test that the desired values are output correctly before using the system with animals!

### 6.3 API

The parameters can be sent to FreiLaser via serial/UART. The parameters are transmitted as a JavaScript Object Notation (JSON) dictionary followed by a line-break character. This enables FreiLaser to operate in fully autonomous mode (Fig. 2**C**), where another system sets the stimulation parameters and controls the mask and trigger signals. Ideally, this system also controls the behavioral task, allowing synchronization of events [22].

Example how to send parameters in Python:

**Figure:**
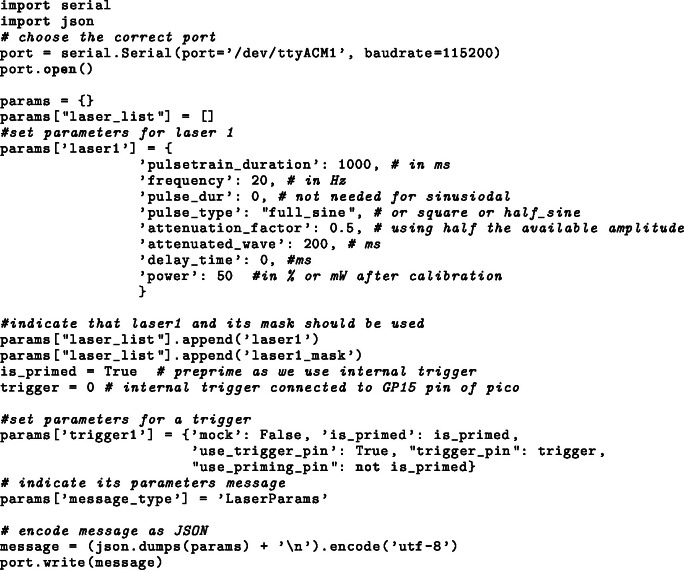

Example how to send software trigger in Python:

**Figure:**
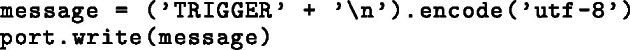

## 7 Validation and characterization

• FreiLaser successfully generated analog and digital signals to control up to four lasers simultaneously.
• FreiLaser is able to generate control signals that fall within the desired range of parameters for optogenetic experiments.
• The generated signals were temporally precise and stable over time.
• FreiLaser was tested and shown to generate square wave signals up to 200 Hz and sinusoidal up to 50 Hz and as low as 0.1 Hz.
• Signals generated by FreiLaser effectively controlled a laser output with the intended parameters.

**Fig. 7:**
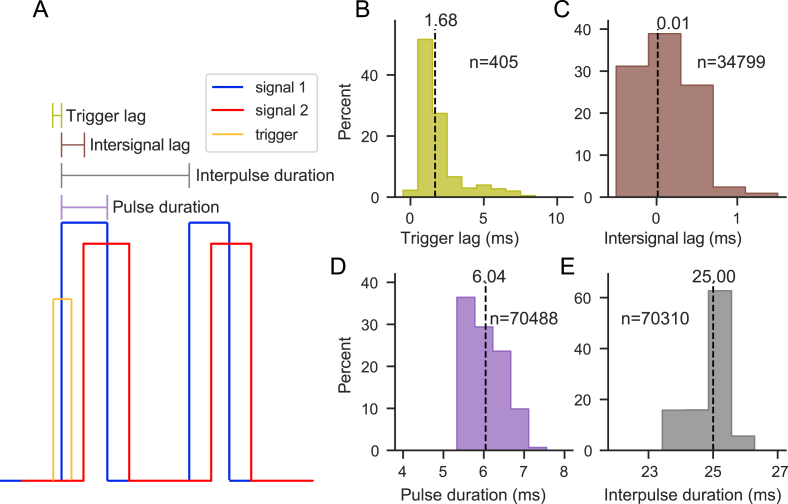
Validation of the timing precision. **A:** Illustration of the tested values. Trigger lag describes the time between the onset of the trigger pulse and the onset of the generated signal. Intersignal lag describes the temporal difference in the onset of individual pulses between the two synched outputs. Interpulse duration describes the time between individual pulses and pulse duration refers to the duration of a single pulse. **B:** Histogram of measured the trigger lag. The average lag of 1.68 ms is well within acceptable range for behavioral experiments. **C:** Histogram of measured offset between two synchronized 40 Hz square wave signals. Signals showed very little offset, with an average of 0.01 ms. **D:** Histogram of measured pulse duration. Average was close to the set duration of 6 ms. **E:** Histogram of measured interpulse duration. The average was 25 ms, as expected for the set frequency of 40 Hz.

To test and validate the FreiLaser system for the intended use case of optogenetic experiments, we recorded the generated signals, trigger, and optical power using a data acquisition system (MCC DAQ 1608G) at 2 kHz (Fig. 7**A**). To validate the timing precision of the FreiLaser, we set the parameters to generate two 25 Hz square-wave signals with 6 ms pulse duration and 10 s total duration. The start of both pulse trains was offset by 200 ms, such that the individual overlapping pulses were expected to coincide. The trigger signal was generated at random time points by an additional Pico board. We determined the trigger lag (Fig. 7**B**) to be approximately 1.68 ms, which is well within the acceptable range for behavioral experiments. The two generated signal trains were almost exactly aligned (Fig. 7**C**), with stable interpulse and pulse durations closely matching the set values. Thus, FreiLaser is able to generate stable, temporally precise signal trains with minimal lag. However, if microsecond (μs) precision (e.g., for spike-triggering) or real-time capabilities are required, alternative systems should be considered.

**Fig. 8:**
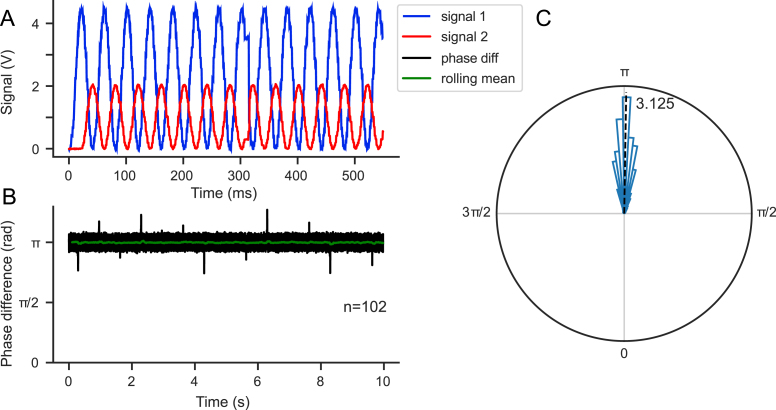
Validation of the analog signal stability. **A:** Overview of the two offset sinusoidal signals. **B:** Phase difference between the two signals. Signals stayed stable over the whole pulse duration. **C:** Circular histogram of the signals phase offset. Average of 3.125 was very close to the expected offset of π.

To validate the stability of the generated analog signal, which is crucial for sinusoidal stimulation, we generated two sinusoidal signals (25 Hz) each with a duration of 10 s, offset by a half-cycle phase shift (π radiants, corresponding to 20 ms) (Fig. 8**A**). We then measured the phase difference between the two signals to confirm their temporal stability over time (Fig. 8**B**) and to verify its alignment with the expected value (Fig. 8**C**). This demonstrates that FreiLaser is capable of generating precise and temporally stable analog signals with sinusoidal waveforms.

**Fig. 9:**
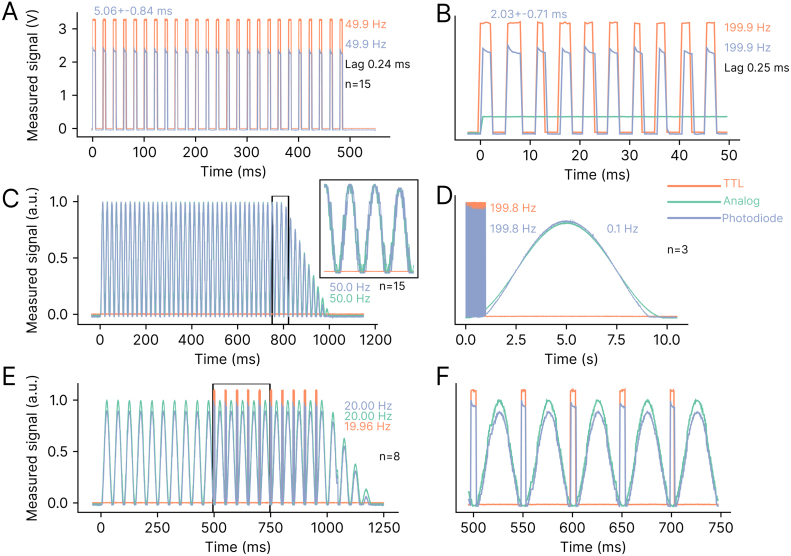
Validation of the laser output control. **A:** Generated control signals and measured optical power(via a photodiode) of the laser output for square wave (50 Hz) stimulation with 5 ms pulses. **B:** Square wave (200 Hz) with 2.5 ms pulses stimulation. **C:** Sinusoidal wave (50 Hz) for 800 ms and 200 ms attenuation. Black rectangle indicates area shown in the insert on the right. **D:** Fast square wave (200 Hz) for 1 s for one laser and simultaneous slow sinusoidal wave (0.1 Hz) for 10 s for second laser. **E:** Sinusoidal wave (20 Hz) for 1000 ms and 200 ms attenuation, paired with 20 Hz square wave stimulation of a second laser. Second laser was delayed by 497 ms to fit between sinusoidal waves. Black rectangle indicates the magnified area in **F:** Magnified segment from **E**.

To validate that the generated control signals could effectively control the output of a laser, we measured the output of an Omicron LuxX 647–140 and Omicron LuxX 473-100 lasers using a Thorlabs photodiode FDS100. The photodiode circuit consisted of a 5 V bias voltage and load resistor value R_l of 1 kΩ. We tested different stimulation types with parameters at, or exceeding, parameters typically used in optogenetic experiments. The laser successfully followed all tested stimulation patterns. FreiLaser could generate precise 50 Hz square pulses (measured freq 49.9 Hz) Fig. 9**A** and even 200 Hz (199.9 Hz measured) Fig. 9**B**. In both cases the measured delay between the control signal and the laser output power was ≈0.25 ms (limited by 2 kHz recording frequency of the DAQ).

For stimulation with modulated analog signals (sinusoidal stimulation), the laser was able to smoothly follow the applied waveform (50 Hz- Fig. 9**C**, 0.1 Hz- Fig. 9**D**, 20 Hz- Fig. 9**E,F**). Furthermore, we demonstrated the ability of the FreiLaser system to modulate multiple lasers simultaneously (Fig. 9**D,E,F**). The validation of the FreiLaser system demonstrated its capacity to generate precise and stable control signals for a range of stimulation patterns, including sinusoidal, half-sinusoidal, and square-wave forms. The reliability and flexibility of the FreiLaser system make it a valuable tool for optogenetic experiments, as it can accurately modulate laser output in complex experimental setups.

## CRediT authorship contribution statement

**Artur Schneider:** Writing – review & editing, Writing – original draft, Visualization, Validation, Software, Methodology, Conceptualization. **Ilka Diester:** Writing – review & editing, Supervision, Funding acquisition.

## Ethics statements

All animal procedures were approved by the Regierungspräsidium Freiburg, Germany (G-20-26). Animal of both sexes were used in the study, however, without any relevance to the results as only hardware validation is presented.

## Declaration of Generative AI and AI-assisted technologies in the writing process

During the preparation of this work the authors used ChatGPT4.o and DeepL-Write in order to improve the readability and language of the manuscript. After using this tool/service, the authors reviewed and edited the content as needed and take full responsibility for the content of the published article.

## Declaration of competing interest

The authors declare that they have no known competing financial interests or personal relationships that could have appeared to influence the work reported in this paper.
